# Maxillofacial Fractures in the Province of Pescara, Italy: A Retrospective Study

**DOI:** 10.1155/2014/101370

**Published:** 2014-01-23

**Authors:** Giuliano Ascani, Francesca Di Cosimo, Michele Costa, Paolo Mancini, Claudio Caporale

**Affiliations:** ^1^Department of Maxillofacial Surgery, Spirito Santo Hospital, Via Fonte Romana 8, 65124 Pescara, Italy; ^2^Department of Otorhinolaryngology, Spirito Santo Hospital, Via Fonte Romana 8, 65124 Pescara, Italy

## Abstract

The aim of the present study was to assess the etiology and pattern of maxillofacial fractures in the Province of Pescara, Abruzzo, Central Italy. Was performed a retrospective review of patients treated at the Department of Maxillofacial Surgery of Spirito Santo Hospital from January 2010 to December 2012. Data collected and analyzed included sex, age, cause of injury, site of fracture, monthly distribution, and alcohol misuse. A total of 306 patients sustaining 401 maxillofacial fractures were treated. There were 173 males (56.5%) and 133 females (43.5%). Most of the patients (36.9%) were in the age group of 18–44 years. The most common causes of injuries were road traffic accidents (26.4%); the second leading cause was interpersonal violence (23.2%), followed by injuries associated with falls (19.2%). Fractures of the mandible (31%) and zygoma (23%) were the most common maxillofacial fractures in our study. The monthly distribution peaked in the summer (July and August, 30.4%) and in October (13.1%). In conclusion, this study confirms the close correlation between the incidence and etiology of facial fractures and the geographical, cultural, and socioeconomic features of a population. The data obtained provide important information for the design of future plans for injury prevention and for education of citizens.

## 1. Introduction

There is a remarkable regional variation in the incidence, sex and age distributions, aetiology, and site distribution of maxillofacial fractures depending upon the geographic conditions, cultural characteristics, and socioeconomic trends [1–6].

The Province of Pescara is a province in the Abruzzo region of Italy; it has an area of 1.187 km² and a total population of 314.391 inhabitants.

Since the Department of Maxillofacial Surgery in the Hospital of Pescara was opened in January 2010 acting as facial trauma centre, no study has been carried out so far to find out the epidemiological data of maxillofacial fractures in our province.

We therefore assessed the etiology and pattern of maxillofacial fractures in patients treated at our centre with the aim to give valuable information for both health care providers and government officials that can be used for the development of public health programs for education and prevention.

## 2. Materials and Methods

We have carried out a retrospective analysis of all patients with maxillofacial fractures surgically treated at the Department of Maxillofacial Surgery of Spirito Santo Hospital, Pescara, Italy, from January 2010 to December 2012.

Data collected and analyzed included sex, age, cause of injury, site of fracture, monthly distribution, and alcohol misuse.

The aetiological factors were classified into road traffic accidents (RTA), interpersonal violence, falls, work- related accidents, and others (iatrogenic, gunshot, pathological, etc.).

In patients with multiple facial bone fractures, each affected bone was evaluated as separate case.

Patients with dentoalveolar fractures and patients with nasal bone fractures were excluded from the study because in our hospital they are treated by dentist and otorhinolaryngologist, respectively.

## 3. Results

A total of 306 patients sustaining 401 maxillofacial fractures were treated at our centre between January 2010 and December 2012.

**Figure 1:**
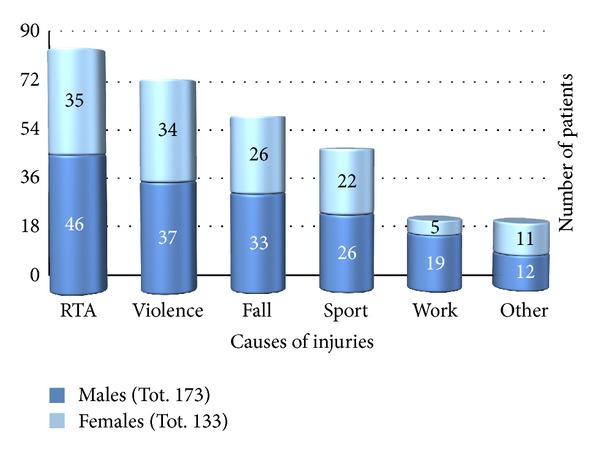
Distribution of patients according to gender and causes of injuries.

There were 173 males (56.5%) and 133 females (43.5%), giving a male to female ratio of 1.3 : 1. Distribution of patients according to gender and causes of injuries is shown in Figure 1.

**Figure 2:**
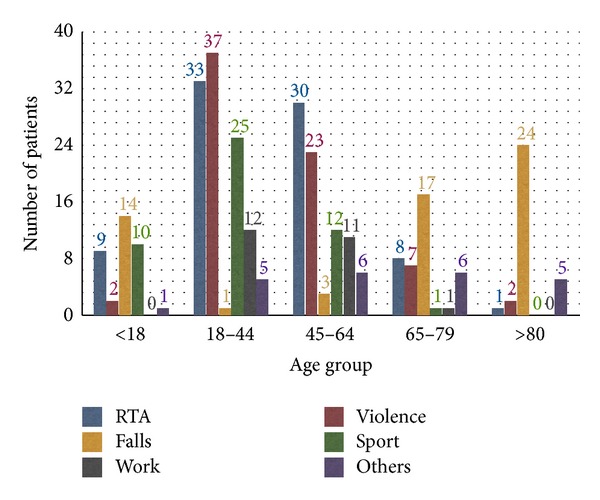
Distribution of patients according to age group and causes of fractures.

Most of the patients (125; 36.9%) were in the age group of 18–44 years, while the smallest number of patients (32; 10.4%) was over the age of 80. Distribution of patients according to age group and causes of fractures is shown in Figure 2.

The most common causes of injuries were road traffic accidents (81 patients; 26.4%); out of these 81 patients, 35 (43.2%) were involved in bicycle accidents, 24 (29.6%) in motorcycle accidents, and 22 (27.2%) in car accidents.

The second leading cause was interpersonal violence (71 patients; 23.2%), followed by injuries associated with falls accounting for 59 (19.2%) of all injuries; out of these 59 patients, 56 (94.9%) injuries were associated with falls on the ground and 3 (5.1%) with falls from height.

Sport-related injuries were documented in 48 (15.7%) patients: 23 playing soccer, 6 rugby, 6 capoeira, 12 bicycle racing, 1 volleyball, and 1 basketball.

Work-related injuries caused maxillofacial fracture in 24 (7.8%) patients.

In 23 (7.5%) patients other causes were recorded: 11 pathological fractures, 8 iatrogenic fractures, 2 collision with a heavy object, 1 hit by horse kick, and 1 gunshot.

Out of the total of 306 patients, 91 (29.7%) presented two or more sites of fractures, average 1.3 fractures per patient; panfacial fractures were treated in 11 (3.6%) patients.

**Figure 3:**
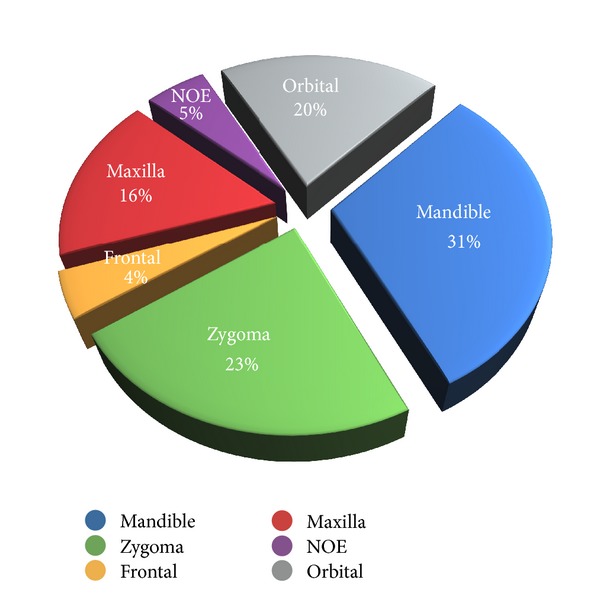
Site distribution of fractures.

Fractures of the mandible (123; 31%) and zygoma (92; 23%) were the most common; site distribution of fractures is shown in Figure 3.

Among the 306 patients, 121 (39.5%) were under the effect of alcohol at the time of injury.

**Figure 4:**
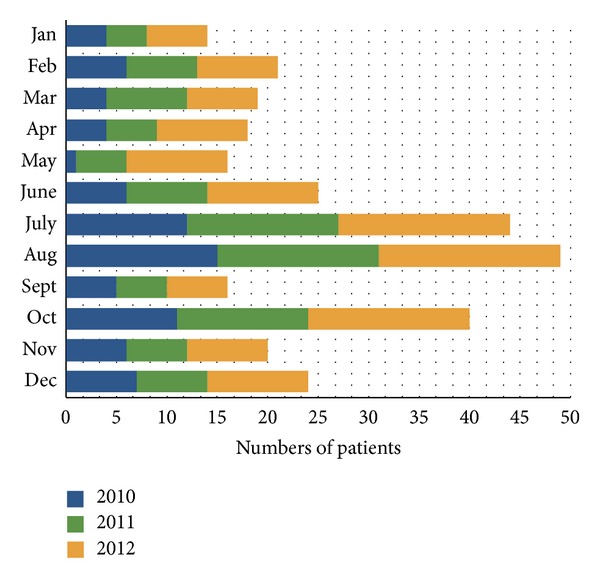
Yearly and monthly distribution of maxillofacial fractures.

The monthly distribution peaked in the summer (July and August, 30.4%) and in October (13.1%); in Figure 4 is shown the yearly and monthly distribution of maxillofacial fractures.

## 4. Discussion

The epidemiological features of maxillofacial fractures are consistently influenced depending on environmental, cultural, and socioeconomic factors, with great variations among populations of different countries and even within the same country [1–7].

Like previous studies, our finding shows that maxillofacial fractures are more common in males, but the male to female ratio in our study (1.3 : 1) was lower than those reported in the international literature [3–8].

In this study, the peak incidence was in the age group of 18–44 years, in agreement with the results of many other authors [3, 4, 9] and reflecting the fact that people in the third decade of life are more active regarding work, sports, violent activities, and high speed transportation.

As throughout the world [3, 9–12] even in our province the primary causes of maxillofacial fractures are road traffic accidents, interpersonal violence, and falls. According to this study, road traffic accidents remain the leading cause of injuries in both males and females, although the numbers of females injured by road traffic accidents and by interpersonal violence are practically equal (35 versus 34) (Figure 1). In contrast to other previous reports [2, 9, 11, 12], violence-related fractures proved to be higher in females (25.6%) than in males (21.4%). In this study, interpersonal violence is the main cause of maxillofacial fractures in patients between 18 and 44 years while falls were the most common cause in children (<18 years) and the elderly (>65 years) (Figure 2).

Among road traffic accidents, two wheelers were responsible for the majority of maxillofacial fractures (59 patients; 72.8% of RTAs); these results were similar to previous studies reported in the literature [2, 3, 10, 11] and can be explained by the fact that bicycles and motorcycles are very popular as means of transport in our province because of its geographical and climatic features.

Among sports-related fractures we reported 6 patients injured by playing capoeira. All of these patients were treated in a period time of 5 months, between September 2011 and January 2012; this is due to the fact that during that period there was a large spread in Italy of capoeira and many people have started to practice this sport. This data confirms the variability over time of the etiology of maxillofacial trauma and the close correlation with the sociocultural trends.

In the present series, the most commonly involved bones were the mandible followed by zygoma. These reports are consistent with studies in several other countries [2–4, 9, 10] but in contrast with one recent study from Italy [13] that indicates the zygoma as the most fractured anatomical site, followed by isolated fractures of the orbital floor.

Our study shows a close association between maxillofacial injuries and alcohol consumption (39.5% of patients); these findings are similar with previous studies from several countries [3, 10, 14, 15]. Alcohol significantly impairs judgment and coordination, facilitates aggression and interpersonal violence, and is a major cause of road traffic accidents.

The monthly distribution of the maxillofacial fractures in our province showed two peaks.

The first one was in July-August and was related to the tourist season during which there is an exponential increase of population in our territory. This was similar to other studies [4, 13]. The second peak was in October and can be explained by the olive-harvesting season. In our province, there is a long tradition of olive oil production that is often a family activity. For this many people, even the elderly and those with little experience, are involved in the olive harvest, resulting in significant number of maxillofacial traumas related to it.

## 5. Conclusions

This study confirms the close correlation between the incidence and etiology of facial fractures and the geographical, cultural, and socioeconomic features of a population.

The data obtained provide important information for the design of future plans for injury prevention and for education of citizens.
